# Management of Gliomas: Overview of the Latest Technological Advancements and Related Behavioral Drawbacks

**DOI:** 10.1155/2015/862634

**Published:** 2015-08-09

**Authors:** L. Ganau, M. Paris, G. K. Ligarotti, M. Ganau

**Affiliations:** ^1^School of Medicine, University of Cagliari, 09124 Cagliari, Italy; ^2^St Thomas Hospital, Guy's and St Thomas NHS Foundation Trust, London SE1 7EH, UK; ^3^Department of Neurosurgery, Niguarda Cà Granda Hospital, 20162 Milan, Italy; ^4^Department of Surgical Science, University of Cagliari, 09124 Cagliari, Italy

## Abstract

The advancements in basic sciences and the availability of sophisticated technological aids to surgical removal of gliomas have led over the last few years to the rise of innovative surgical strategies, the identification of better prognostic/predictive biomolecular factors, and the development of novel drugs and all are meant to profoundly impact the outcome of patients diagnosed with these aggressive tumours. Unfortunately, the treatment protocols available nowadays still confer only a small survival advantage at a potentially high cost in terms of overall well-being. In this review we identified the potential and limits of the most promising research trends in the management of glioma patients, also highlighting the related externalities. Finally, we focused our attention on the imbalance between the technical and behavioral aspects pertinent to this research area, which ultimately represent the two sides of the same coin.

## 1. Background

Gliomas represent the most frequent class of malignant primitive tumours of the central nervous system (CNS). According to their aggressiveness, the World Health Organization (WHO) classifies them into Grades 1 and 2 or low-grade gliomas (LGG) and Grades 3 and 4 or high-grade gliomas (HGG).

Although relatively rare (incidence of 5/100,000 persons/year in Europe and North America), HGG are associated with disproportionately high morbidity and mortality regardless of the application of state-of-the-art treatment strategies; in fact, their outcome remains poor, with a median survival of only 14.6 months [1]. Also LGG, despite the relatively slow growth, do not present a good outcome, as approximately 70% of Grade 2 gliomas are known to evolve to anaplasia, leading to neurological disability and ultimately to death within 5–10 years [2]. Furthermore, both LGG and HGG are characterized by a wide clinical and histological heterogeneity; this is particularly true for HGG, because 35–40% of them have epigenetic modifications as the underlying mechanism driving malignancy [3]. As a result, scientists and clinicians all over the world still fail in predicting the clinical evolution of each single patient diagnosed with those CNS neoplastic lesions. Thus, the neurooncology community has tried to put all possible efforts in the identification of better diagnostic and therapeutic strategies aimed at improving the prognosis of those patients.

In particular, the advancements in basic sciences and the availability of sophisticated technological aids to surgical removal of those tumours have led over the last few years to the identification of better prognostic/predictive biomolecular factors, the development of novel drugs, and the rise of innovative surgical strategies, all meant to profoundly impact the outcome of patients harboring gliomas. On the other hand, the significant technological leap has provided those patients with only minimal benefits, mostly in terms of survival rather than in terms of quality of life (QoL).

This review article aims at (1) identifying the potential and limits of the most promising research trends in the management of glioma patients, also highlighting the related externalities, and (2) focusing our attention on the imbalance between the technical and behavioral aspects pertinent to this research area, which ultimately represent the two sides of the same coin.

## 2. Standard of Care

The current mainstay for treatment of HGG is maximal resection (ideally, gross total resection: >95% of the lesion) followed within 30 days from surgery by radiation therapy with concurrent or adjuvant chemotherapy [4]. Clinical evidence that a proactive and aggressive treatment plan improved the outcome of glioma patients when compared to biopsy alone prompted maximum but safe resection to become the ultimate goal of the neurosurgical treatment [4, 5]. Nonetheless, the improvement of patient outcome following gross total resection of HGG mainly relies on extended progression-free survival, rather than on improved QoL or overall survival. Specifically, the impact of surgery on progression-free survival and overall survival has been debated for decades, because of the contradictory results published prior to the diffusion of routine postoperative MRI and volumetric analysis of the extent of resection, which ultimately confirmed the importance of radical removal to extend survival [6].

Concerning LGG, some recent studies have shown similar results confirming that LGG managed with biopsy or subtotal resection followed by a wait and see approach have a higher risk of malignant transformation compared to those treated with more extensive tumour removal [7, 8]. Other studies suggested that the extent of removal does not influence the outcome, whereas the best improvements generally come from the postoperative gain of previously impaired functions. To this regard, Talacchi et al. concluded that although the worsening in executive functions soon after operation leaves the overall cognitive burden initially unchanged, it is often transitory and capable of improvement prospectively [9].

In the process to enhance the efficiency of surgical resection, adjuvant radiotherapy has established itself as one of the most valuable tools, especially because conformational treatment now allows preserving the surrounding healthy brain parenchyma. A recent study meant to establish the role of adjuvant radiotherapy on 14.461 patients affected by HGG found a statistically significant interaction between overall survival and histological grade, whereas no significant interactions were observed between radiotherapy and extent of resection [10]. The effect of radiotherapy is further increased by adjuvant chemotherapy with the alkylating/DNA methylating agent temozolomide. This treatment strategy known as Stupp protocol is nowadays the gold standard regimen for Grade 4 gliomas, as it demonstrated providing patients with a significant increase in 2-year survival from 10.4% to 26.5% [11].

Whilst the adjuvant radio- and chemotherapy options have become established treatment modalities for HGG, their role in LGG is highly debated. Several studies have been conducted over the years on this topic, and recently some long-term survival analyses are becoming available. For instance, Nitta et al., aiming to test the hypothesis that adjuvant therapy might not be necessary for LGG cases in which total radiological resection is achieved, enrolled in a longitudinal study a total of 153 patients treated for LGG between 2000 and 2010 [12]. The multivariate analysis conducted on the data retrieved from those patients did not identify MIB-1 index or radiotherapy as prognostic factors, but it did identify chemotherapy as a prognostic factor for progression-free survival, and extent of resection for both progression-free survival and overall survival of LGG [12]. These findings support the currently most accepted practice of using more aggressive treatment with radiotherapy only in LGG patients with a poor prognosis, such as those with diffuse tumours (in particular astrocytomas rather than oligodendrogliomas) and those with partial resection.

## 3. Technical Advancements, Potentials, and Limits

Because maximum but safe resection represents the main goal in terms of neurosurgical management of both HGG and LGG, the surgical and medical treatment of deep-seated or functionally critically located tumours has become its ultimate frontier.

### 3.1. Intraoperative Imaging and Navigation

Whenever gliomas are located in critical brain areas, as those responsible for speech or motor functions, aggressive surgical excision might not be efficiently and safely feasible, requiring instead a tailored management strategy. Recently, different technical developments for intraoperative tumour visualization, navigational systems, or intraoperative magnetic resonance (MR) imaging have been introduced into daily practice, and other techniques for radiological planning and robotic neurosurgery are in advanced stages of laboratory validation [13]. In particular intraoperative neuronavigation, along with cortical and subcortical electrostimulation (IOM), or awake surgical techniques have provided the operating neurosurgeon with a continuous feedback to estimate the extent of resection [4]. Though, despite the use of neuronavigation the rates of complete resection in resectable tumours are disappointingly low both in LGG and HGG and range from 20% to 60% [6]. Also, patients with large tumours are more prone to worsening in executive functions soon after operation, despite the use of IOM [9].

More accurate imaging techniques might play a role in tumour identification, and the key to this is pre- and intraoperative contrast enhancement; thus significant efforts are put in the characterization of compounds that have a high affinity in binding to glioma cells leading to an optimized definition of its boundaries. Unfortunately, researches in the field of nanoparticle-based contrast agents are still in the preclinical state; nonetheless their premises are outstanding because of their potential selective accumulation within the tumour (instead of diffusing freely into and out of a tumour like common agents do) as their retention effect would result not only from the tumour's innate altered vascular architecture but also from the expression of neoplastic vascular mediators of extravasation [14]. In the meantime, the altered tissue metabolism is already offering a valuable ploy: HGG in fact may be intraoperatively detected thanks to the orally administered drug 5-aminolevulinic acid (5-ALA) leading to fluorescence of tumour cells during surgery, allowing identification and resection of tissue that is otherwise indistinguishable from the brain parenchyma [15].

### 3.2. Trends in Chemotherapy

Furthermore, research is also shifting into investigating the complex cellular and molecular glial tumour-genesis responsible for the recurrence of the disease. These concepts have led to envisaging the next generation of chemotherapy agents, which might activate immunologic memory serving as vaccines directed towards gliomas' specific antigens [28] or might act as veritable Trojan horses meant to specifically target tumoural stem cells and circulating tumour cells [29].

In fact, beside the great success of temozolomide, the newer drugs now available on the market are failing to deliver the expected results in terms of extended progression-free survival or overall survival; on the other end QoL is being significantly affected by their toxicity. An example of the limited efficacy of second line chemotherapy strategies is represented by antiangiogenic agents. Bevacizumab, the most known of this class of drugs, has unfortunately shown determining only an increased incidence of pseudoresponse with almost no impact at all on overall survival [6]. Following antiangiogenic therapy, the rapidly decreasing extent of contrast-enhancing tumour volume is in fact due to transitory restoration of the blood-brain-barrier rather than a more consistent antineoplastic activity [6].

### 3.3. Radiotherapy and Radiosurgery

Noteworthy, some advancements have also been accomplished in the field of radiation therapy. For instance, the observation that local control and median survival can be improved through the radiation dose escalation has gradually introduced stereotactic radiosurgery (SRS) in the therapeutic panel for HGG. Although limited data are available concerning salvage SRS, a 2012 retrospective study of 77 recurrent HGG patients showed that the median posttreatment survival doubled for those receiving Gamma Knife SRS compared to patients treated with second surgery alone and advocated SRS as an alternative to open surgery for HGG at the time of recurrences, because of the significantly lower complication rate [30].

To this extent, as anticipated above, LGG represent a substantially different issue: here oncotypes may be considered “late responding” radiobiological targets due to their relatively small proportion of cells in a proliferative phase of the cell cycle [31, 32]. In a recent clinical trial, using a protocol for fractionated SRS an 83% rate of partial or complete tumor regression and 11% of stabilization of disease were achieved [33]. Progression-free survival was 92% at 3 years and 88% at 5 years, but moderate acute or late toxicity was observed in 5% of patients [33]. Therefore SRS seems to combine the effectiveness of the conformal 3D dose distributions with a reduced toxicity of fractionation; however, given the persistent possibility of malignant transformation of these lesions and cognitive impairment this therapeutic option remains debated [34]. Hopefully, the scientific contribution of several clinical trials aimed at better defining the radiobiological parameters of HGG and LGG is recently leading to some attempts to create tumor control probability models meant to revolutionize in the near future the radiotherapic and radiosurgical protocols that we are currently using in the clinical practice [32, 35].

### 3.4. Genomics and Proteomics of Gliomas

Finally, it is becoming more and more evident that the failures of current management strategies are due to the poor understanding of the many biological variables responsible for the phenotypic behavior of those tumours, their aggressiveness, and their tendency to recur locally despite complete resection followed by radiotherapy and chemotherapy treatments. Imaging techniques based on nuclear medicine and many candidates for metabolite signature are under investigation, and they might allow identification of neoplastic spreading into normal brain in regions that do not appear abnormal on standard MRI, as well as distinction between pseudoprogression (nontumoural postoperative radiological changes) and disease recurrence [36].

Such biomolecular markers would be of great value to neurosurgeons, radiation oncologists, and neurooncologists in optimizing brain tumor treatment. Several nanodevices for single cell proteomic analysis are also in advanced stage of investigation with potential to shed light on the fingerprint of gliomas [36]. They all come with great expectations and in the near future might prove to be extraordinary tools providing new insights into the biological understanding of HGG and LGG upon which enhanced therapeutic strategies are based. In fact, the development of novel therapies will only come from an unprecedented integration of neuroscience, bioengineering, molecular biology, and physiology, enabling a real personalization and adjustment of treatment, which are critical for patients with gliomas in whom all existing therapies, sooner or later, are expected to fail.

## 4. Behavioral Aspects and Neurophysiological and Neuropsychological Perspectives

Behavioral neurology has benefited from numerous basic science investigations and clinical trials on LGG and HGG, especially in the field of neuroplasticity. Neuroimaging and IOM studies on glioma patients have led to a better understanding of the crucial role played by associative cortex and white matter in information processing and brain function.

Some elegant examples, highlighting the latest breakthrough in areas as diverse as consciousness, verbal expression, and empathy, deserve mention. Herbet et al. recently showed for the first time the importance of medial posteroparietal cortex (PPC) in conscious information processing: through their data on IOM they demonstrated that the functional integrity of the PPC connectivity is necessary for maintaining consciousness of external environment [37].

Given the paramount importance during awake surgery several groups investigated the connectivity at the base of the phonological system. Nomura et al. showed how the dominant uncinate fasciculus contributes to semantic memory and naming performance by outlining that during IOM direct electrostimulation of this fasciculus may disrupt its crosstalk with the memory circuit resulting in naming difficulty, verbal paraphasia, and recurrent and continuous perseveration [38].

Maldonado et al. showed that the supramarginal gyrus, connected to the ventral premotor cortex by horizontal fibers of the superior longitudinal fascicule, subserves articulatory processing, as demonstrated by dysarthria elicited during IOM [39]. Furthermore they highlighted the role of the long arcuate fibers, found deeper in the white matter, in phonological processing, as supported by phonemic paraphasia induced by electrostimulation, and also confirmed that no semantic disturbances result from stimulation of the superior longitudinal fascicule, including its posterior part [39].

Finally, studies on LGG have also shed light on the human empathic experience, which is a multifaceted psychological construct, characterized by both subjective and cognitive aspects, arising from functional integration of multiple neural networks. In fact, despite accumulating knowledge about the cortical circuitry of empathy, until recently almost nothing was known about the anatomical and functional connections conveying empathy-related neural information. This research sector has already led to some fundamental neuroradiological and neurophysiological evidences: thus it is now known that the disconnection of the left cingulum bundle in glioma patients represents a strong predictor of a low cognitive empathy; similarly the involvement of the right uncinate fasciculus and the right inferior frontooccipital fasciculus is related to a low and a high subjective empathy, respectively [40].

Those are just few good examples of how studies on gliomas have provided interesting insights into the pathophysiology of other diseases, such as those conditions characterized by abnormalities of long-range anatomical connectivity like coma, dyslexia, autism, schizophrenia, and dementia. Despite all the positive externalities, the favorable repercussions of those research achievements are still dismal for glioma patients; in fact while the minimal improvements in progression-free survival and mortality are just one aspect of the problem, they nonetheless provide a measure of the mean impact that research is still having on current treatment strategies.

As shown above, in order for treatments to be more effective, clinicians are pushing the limits towards greater aggressiveness and radicality, and this process is also accompanied by numerous drawbacks with significant neurological, psychological, and economical burden [41]. The real question is therefore the following one: in our daily battle to fight those CNS tumours are we eventually scotomizing what really matters to our patients?

The concept of patient-centered medicine implies that patient-reported outcome and the related behavioral aspects are as important as the objective neurological examination and performance scales [42]. To this regard it is well known that any CNS malignancy has numerous deleterious effects on patient's QoL and well-being; and gliomas in particular are known to cause several cognitive deficits in many functional domains such as intelligence, executive functions, memory, language, praxis, gnosis, and mood state.

Rooney et al. reviewed a total of 42 observational studies of depression in glioma patients and found a median prevalence of 28% compared to 2–4% in the general population [43]. Habets et al. reported that up to 79% of glioma patients present cognitive impairments undermining their independence in the activities of daily living [44]. Those are now emerging clinical issues as the general lack of comprehensive neuropsychological assessment performed preoperatively and in the acute postoperative period has prompted neurosurgeons to reconsider the need for cognitive assessment in the course of treatment [9]. Landmark papers published over the last 5 years on this topic are reported in Table 1 for HGG [16–22] and Table 2 for LGG [23–27]. The progressive, almost exponential, growth in terms of papers published on QoL is undoubtedly the best parameter to understand the increased interest in the neurooncology community toward this argument.

## 5. Tailoring the Decision Making Process

In light of the status quo depicted above one last topic deserves particular attention: the individual decision making process on who to treat and how to treat. Gliomas oblige patients to deal with the anxiety-provoking perspective of local recurrence of the disease, reoperations, postoperative deficits, and treatment-related side effects. On one hand, some studies are demonstrating that patients more satisfied with respect to decisional involvement seem able to better cope with their disease and show a significant better self-perceived QoL [45]. On the other hand, research evidence, as showed above, is often poor, inconclusive, or fragmented; and doctors seeking answers in the most up-to-date scientific literature often have troubles in orientating themselves among preliminary results, experts' opinions, and controversies. Astonishingly, management choices may differ widely even in a relatively homogeneous group of specialists [46]. As a consequence, it is important to reconsider how specific treatment decisions are taken, especially in an era more and more oriented toward the goal of personalized medicine. Providing sensible information and disclosing all the pros and cons of each single treatment option are technically and emotionally demanding and time consuming and may pose special challenges to the process of requesting and obtaining a fully informed consent. This aspect is now supported by clinical evidence; in fact Triebel et al. in a case control study demonstrated that the capacity to consent to treatment tested with standardized psychometric questionnaires is impaired soon after diagnosis in more than 50% of HGG patients compared to healthy controls [47]. Similar findings were published by Marson et al.; specifically they showed that the enrollment of glioma patients in prospective clinical trials may raise ethical issues, as 23% to 38% of patients with HGG show after diagnosis impairments in research consent capacity [48]. Finally, we must always bear in mind that the clinical status of glioma patients, especially HGG ones, is subject to sudden deterioration: as reported by Sizoo et al. more than half of those patients become incompetent relatively early to make decisions due to delirium, cognitive deficits, and/or decreasing consciousness obliging doctors and caregivers to shift the goal of therapy from primarily life-prolongation to primarily sustaining the QoL [49]. As such, pragmatically recognizing that second opinions and multidisciplinary meetings must play a pivotal role in rationalizing the management of HGG and LGG becomes today more and more valuable to ensure that each patient receives the best course of treatment.

## 6. Conclusions

The technological advancement witnessed in the treatment strategies nowadays available for HGG and LGG is still conferring a relatively small survival advantage at a potentially high cost to the overall well-being of the patients. In this review we have highlighted some reasons for this imbalance between potential technical aids and the behavioral aspects pertaining to those tumours. This work has helped to draw some considerations: (1) although new molecular pathways crucial to the biology and invasive ability of gliomas are coming to light, the pace of effective translation from bench to bedside of the latest basic science achievements is unfortunately slow; (2) the lack of well-designed randomized clinical trials upon which clinical decisions regarding the most appropriate and safe surgical, radio- and chemotherapeutic management options are based, still represents the main limiting factor for the establishment of internationally accepted guidelines for both HGG and LGG; (3) a shift towards personalized medicine seems the most promising approach to those patients.

For those reasons, on one hand it is important to emphasize the need for basic research and multicenter randomized clinical trials; on the other hand the recommendation, for the time being, should be that doctors compassionately approach each case on an individual base and weigh the risks and benefits of every possible management strategy directly with the patients and his/her next of kin. In this process a specific point that must be carefully taken into account is the patient's psychological sphere. Moreover, as his/her pathological conditions and related objective/subjective expectations may change rapidly along the course of treatment, a constant communication path has to be established among doctors, patients, and caregivers. Unfortunately, this is the best safe net currently available for thousands of patients diagnosed every year with this aggressive class of primary brain tumours.

## Figures and Tables

**Table 1:** QoL studies on HGG.

Reference	Study design/topic (number of patients)	Findings
Yavas et al., 2012 [16]	**Prospective cohort** Predictors of progression **(118)**	Emotional function, insomnia, appetite loss, future uncertainty, and communication deficit significantly relate to disease progression

Jakola et al., 2011 [17]	**Prospective cohort** Predictors of survival **(61)**	Early deterioration in QoL after surgery is linked to overall survival and reflects both the burden of symptoms and treatment hazards

Sizoo et al., 2014 [18]	**Retrospective cohort** QoL at the end of life caregivers perspective **(83)**	Cognitive, physical, and psychological functioning deteriorate over time; acceptance of disease increases slightly towards death. Support from social environment and dying with dignity are important determinants of QoL

Sagberg et al., 2014 [19]	**Prospective cohort** Response to therapy **(164)**	QoL questionnaires are responsive to changes when glioma patients are deteriorating functionally after surgery but not responsive when patients are improving

Pompili et al., 2014 [20]	**Retrospective cohort** Palliative care and end of life issues **(122)**	Positive cost-effectiveness of a well-trained neurooncology team managing neurological deterioration, clinical complications, rehabilitation, and psychosocial problems with a multidisciplinary approach

Peters et al., 2014 [21]	**Prospective cohort** QoL and recurrences **(237)**	Fatigue is a strong independent predictor of survival that provides incremental prognostic value to the traditional markers of prognosis in recurrent HGG

Halkett et al., 2015 [22]	**Survey analysis** Predictors of distress **(116)**	Poor function, lower education, and limited financial resources may help identify patients requiring additional screening, information, and psychological support

**Table 2:** QoL studies on LGG.

Reference	Study design/topic (number of patients)	Findings
Aaronson et al., 2011 [23]	**Retrospective** **case control** Cognitive deficits **(195)**	Epilepsy burden and neurocognitive deficits rather than time since diagnosis, tumor lateralization, extent of surgery, and radiotherapy show a consistent relationship with QoL

Yavas et al., 2012 [24]	**Prospective cohort** Response to therapy **(43)**	Function scores return to baseline after active treatment in all patients but those who use antiepileptic drugs

Giovagnoli et al., 2014 [25]	**Survey study** QoL and disease phase **(291)**	Affective well-being is predicted by the phase of disease, while self-perception and confidence are independent of tumor progression and treatment

Jakola et al., 2014 [26]	**Retrospective cohort** QoL and surgery **(79)**	In long-term survivors an aggressive surgical approach does not lower QoL compared to watchful waiting

Nwachukwu et al., 2015 [27]	**Retrospective cohort** Pediatric population **(121)**	Patients with tumor recurrence reported significantly lower role functioning, social functioning, and more financial problems compared to their counterparts
